# Hydrogeochemistry Evidence for Impacts of Chemical Acidic Wastewater on Karst Aquifer in Dawu Water Source Area, Northern China

**DOI:** 10.3390/ijerph18168478

**Published:** 2021-08-11

**Authors:** Henghua Zhu, Jianwei Zhou, Zhizheng Liu, Lizhi Yang, Yunde Liu

**Affiliations:** 1 School of Environmental Studies, China University of Geosciences, Wuhan 430074, China; hhzhu2008@sina.com (H.Z.); jw.zhou@cug.edu.cn (J.Z.); 2 Shandong Institute of Geological Survey, Jinan 250000, China; liuzhizheng1985@126.com (Z.L.); yanglizhi23@163.com (L.Y.)

**Keywords:** groundwater, karst aquifer, hydrogeochemistry, groundwater quality, pollution

## Abstract

The study of the hydrochemical characteristics and the water–rock interaction of karst groundwater is very important for the rational exploitation of karst groundwater and its pollution control. In this paper, the systematic clustering method was used to analyze the hydrochemical characteristics of different types of groundwater, combined with hydrochemical graphic analysis and correlation analysis to explore the impact of chemical acidic wastewater on the evolution of karst aquifer in the Dawu water source area, northern China. The results show that the chemical acid wastewater, sourcing from discharges/spillages from the local chemical industries, has different degrees of pollution impact on karst groundwater, causing the total hardness of all karst groundwater and the total dissolved solids, Cl^−^ and SO_4_^2−^ in nearly half of the karst groundwater to exceed the quality indexes of class III water in China’s standard for groundwater quality (GB/T 14848-2017). Hydrochloric acid and sulfuric acid in the wastewater can be buffered by the dissolution of carbonate rocks, resulting in a nearly neutral pH (pH-buffering effect) and an increase in Ca^2+^, Mg^2+^, Sr, Cl^−^ and SO_4_^2−^ concentrations in karst groundwater.

## 1. Introduction

Karst groundwater provides an important water resource guarantee for social and economic development. Karst groundwater provides drinking water for 10% of the world’s population, and is an indispensable resource for ecosystems, agriculture and groundwater-dependent activities [1,2]. However, the sustainable utilization of karst groundwater is facing challenges in terms of both quantity and quality, since karst aquifers are extremely vulnerable to climate and anthropic pressures [3,4,5,6]. Karst groundwater resources are particularly vulnerable to contamination resulting from intense agriculture and other anthropogenic activities, and the quality of karst groundwater continues to deteriorate, resulting in water quality-based water shortages in karst regions [7,8,9,10]. For example, the concentrations of total dissolved solids (TDS), total hardness (TH), SO_4_^2−^ and Cl^−^ are increasing continuously, which is one of the main manifestations of karst groundwater quality deterioration [11,12,13,14].

The TDS, total hardness, SO_4_^2−^ and Cl^−^ in uncontaminated karst groundwater are primarily determined by the dissolution–precipitation of carbonates and intercalated evaporating salt (gypsum and halite) in karst aquifers, and their concentrations are relatively stable or slowly changing [15,16,17]. Anthropogenic inputs significantly change the chemical composition of groundwater, resulting in the significant increase in TDS, total hardness, SO_4_^2−^ and Cl^−^, etc. Previous studies mainly focused on the hydrochemical response of karst groundwater to acid mine drainage, agricultural activities and acid atmospheric deposition [18,19,20,21]. However, little work has been conducted on the impacts of chemical acidic wastewater on groundwater hydrogeochemistry in karst aquifers. We can speculate that chemical acid wastewater pollution not only directly inputs pollutants into karst groundwater, but also accelerates carbonate dissolution, thus increasing Ca^2+^, Mg^2+^ and HCO_3_^−^ in karst groundwater, and leading to the continuous increase in total hardness. Therefore, it is of great significance to carry out an impact study of chemical acid wastewater on karst groundwater.

The Dawu water source area is a super-large fracture-karst water source in Northern China [22,23]. Since exploitation of the Dawu water source began in 1960, it has provided a reliable and secure domestic water supply for the city of Zibo and large state-owned industrial enterprises in the Linzi area, e.g., Sinopec Qilu Petrochemical Company [24]. For historical reasons, many petrochemical and chemical enterprises such as oil refineries, ethylene plants, fertilizer plants and chlor-alkali plants have been built in the limestone area around the water source area, which has resulted in serious groundwater contamination [24,25,26,27,28]. Hougao, in particular, is the most seriously polluted area of the Dawu water source, with the highest concentration of chloride exceeding 1000 mg/L according to previous water quality monitoring data [24,29]. A large number of chemical enterprises are distributed in the Hougao area; these use inorganic acids such hydrochloric acid as raw materials and produce chemical acid wastewater. There are risks that inorganic acids and acidic wastewater could be lost to the environment through leakage during production, storage and transportation, thereby posing a great threat to the groundwater safety of the Dawu water source. However, to date, the impacts of chemical acidic wastewater on karst aquifers in the Dawu water source area are unclear.

Thus, Hougao, as part of the Dawu water source area, was selected as the study area for this paper, and the water quality and the hydrochemical characteristics of karst groundwater were analyzed. The origin and control process of the main inorganic components (SO_4_^2−^ and Cl^−^, etc.) in the karst groundwater were clarified, and the impacts of chemical acidic wastewater on karst aquifer were revealed.

## 2. Materials and Methods

### 2.1. Study Area

The study area is located in the southwest of Linzi City, belonging to the water-rich area of Dawu, which includes Liuhang, Hougao, Xixia-Dawu, etc. The area is in a warm temperature region with a continental monsoon climate. The annual average temperature is 12.5 °C and the annual average rainfall is 640.5 mm, mainly falling as rain in the summer.

In the south, the low mountains, hills and valleys and the exposed rocks are Ordovician limestones. The northern area is a piedmont inclined plain, with the plain is covered by Quaternary sediments. The water-bearing rocks are mainly the pore water-bearing rocks of the upper Quaternary loose rocks and the fissure karst water-bearing rocks of the lower Ordovician carbonate rocks [24,30]. The groundwater exploitation is mainly concentrated in the carbonate fractured karst aquifer. The karst aquifer is mainly recharged by precipitation in the southern mountainous and the leakage from the eastern Zihe River [24]. Karst groundwater generally flows northward and northeastward until it is blocked by the Carboniferous-Permian coal-bearing strata in the north, forming a huge underground reservoir with an exploitation volume accounting for about 350,000 m^3^/day [30].

### 2.2. Sampling and Analytical Methods

**Figure 1:**
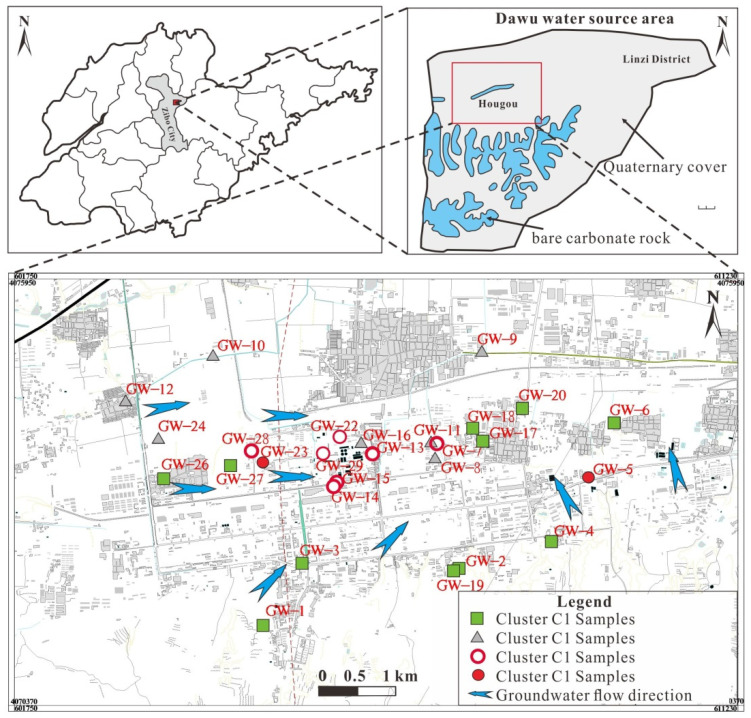
Spatial distribution of groundwater samples with different cluster types.

A total of 27 groundwater samples were collected in July 2018, and the sampling locations are shown in Figure 1. Sampled wells were selected to represent different degrees of pollution as well as groundwater flow direction. In situ field measurements were made on water samples for water temperature (T), electrical conductivity (EC) and pH. Prior to sample collection, groundwater was pumped from the well until the temperature, EC and pH became constant. All sampling bottles were rinsed with sample water three times before collection of the sample for analysis. HCO_3_^−^ concentrations in all groundwater samples were determined using the alkalinity titration method within 1 day. Samples for cation and anion analysis were filtered on site using PTFE-membrane filters (0.45 µm). Filtrate for cation analysis was transferred into 50-milliliter PET sample bottles and immediately acidified to pH < 2 by the addition of HNO_3_ (GR grade). Samples for anions analyses were filtered into 50-milliliter PET sample bottles without preservation. All the cation and anion samples were stored in an ice chest at a temperature of 4 °C and later transferred to the laboratory and stored in a refrigerator at a temperature of 4 °C until analyses. Anions (Cl^−^, SO_4_^2−^ and NO_3_^−^) were measured using ion chromatography (IC), and cations (Ca^2+^, Mg^2+^, K^+^, Na^+^ and Sr) were measured using Inductively Coupled Plasma Atomic Emission Spectrometry (ICP-OES). The accuracy of the chemical analysis was verified by calculating ion-balance errors, where the errors were generally around 10% [31,32], which is an acceptable error for the purpose of this study.

## 3. Results and Discussion

### 3.1. Cluster Analysis

**Figure 2:**
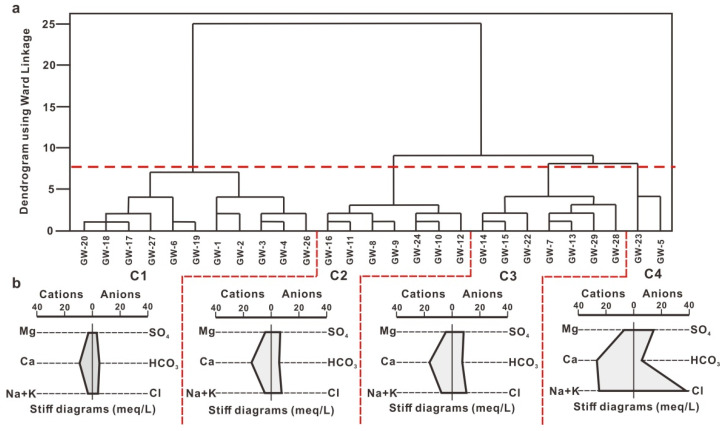
Dendrogram of the Q-mode hierarchical cluster analysis (**a**) and Stiff diagram showing the average ion compositions of each cluster (**b**).

Cluster analysis can classify samples according to their affinity and similarity and can effectively separate different types of samples, which is helpful to study the chemical characteristics and evolution rules of groundwater. In this paper, groundwater samples in the study area were grouped using Q-mode hierarchical cluster analysis using Ward’s minimum variance algorithm and Euclidian distance [33,34]. To make the data closer to normality and remove the impacts of data units on the statistical analysis, log-transformation and a standardization (z-score) of the hydrogeochemical data was performed and used as the cluster variables [33,34]. Figure 2 shows the dendrogram of underground water samples in the study area. Groundwater samples were divided into four clusters of the groundwater samples (namely C1, C2, C3 and C4), according to the phenon line, with a linkage distance of 7.5 (Figure 2).

The spatial distribution of groundwater samples of different cluster types is centered within the area defined by C3 samples, as shown in Figure 1. C3 samples were mainly collected at Hougao and its environs where groundwater in the study area was collectively discharged due to artificial exploitation. In order to ensure the safe supply of groundwater taken from Dongfeng-Xixia section by the Zibo urban water supply company, groundwater in the Hougao section was strongly exploited all year round, resulting in a lower water level than that in the Dongfeng-Xixia section, thereby preventing the spread of pollution from the Hougao section to the East. C1 samples were mainly distributed in the southwest, south and east regions, while C2 samples were mainly distributed in the northern region, both of which were in the groundwater recharge-runoff area. It can be seen from the stiff diagram (Figure 2b) that the main hydrochemical ion contents (Ca^2+^, Na^+^, Cl^−^ and SO_4_^2−^) of C1 samples are significantly lower than those of C2 samples, and both hydrochemical ion contents in C1 and C2 are lower than those of C3 samples at the same time, which indicates that hydrochemical ion content depends on the recharge-runoff conditions of groundwater and the impact of anthropogenic activities in the study area. There are only two samples in the C4 category, and they are remote from one another on the scale of the sample area being located to the east (GW-5) and west (GW-23) of Hougao.

### 3.2. Hydrochemical Characteristics of Different Types of Groundwater

**Figure 3:**
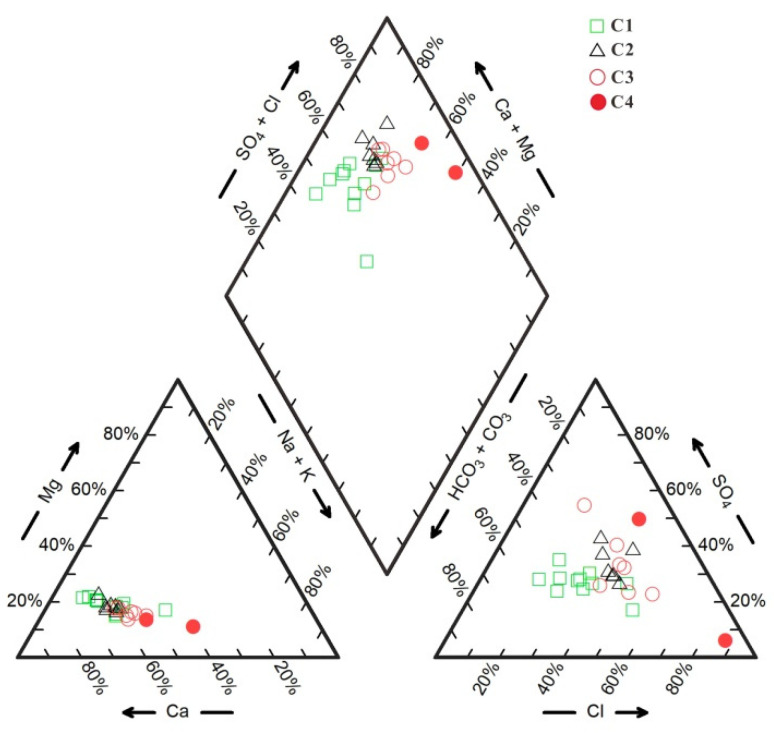
Piper three-line diagram of groundwater hydrochemistry in the study area.

The hydrochemical types of groundwater in the study area are shown in the Piper three-line diagram (Figure 3), and there are significant differences among different cluster categories, which, in turn, indicates that the above clustering analysis is effective in separating different types of samples. The main cation in the C1 and C2 samples is Ca^2+^, and the differences between the hydrochemical types are mainly reflected in the anions. In particular, the hydrochemical types of C1 samples are mainly HCO_3_·Cl·SO_4_-Ca, HCO_3_·Cl-Ca and HCO_3_·SO_4_-Ca, while the hydrochemical types of C2 samples are mainly Cl·HCO_3_·SO_4_-Ca and SO_4_·Cl·HCO_3_-Ca. The hydrochemical types of C3 samples are complex, including HCO_3_·Cl-Ca·Na, Cl·HCO_3_-Ca·Na, Cl·SO_4_·HCO_3_-Ca·Na, SO_4_·Cl·HCO_3_-Ca·Na, Cl-Ca·Na, etc. The hydrochemical types of C4 samples are SO_4_·Cl-Ca·Na and Cl-Na·Ca. In general, along the direction of groundwater runoff (from C1 and C2 to C3), the milligram equivalence percentages of Ca^2+^ and HCO_3_^−^ decrease, while those of Na^+^, Cl^−^ and SO_4_^2−^ increase.

**Figure 4:**
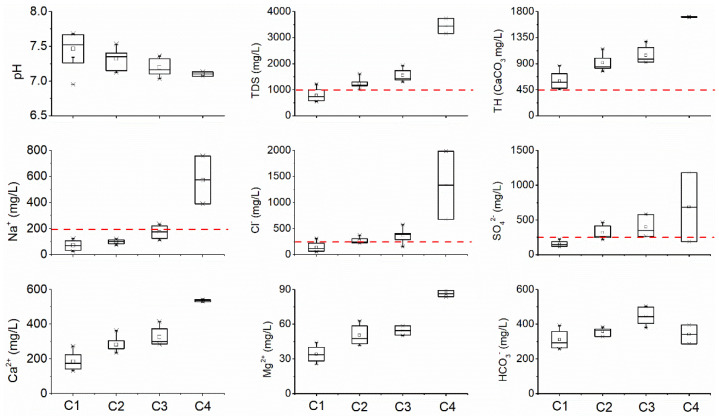
Box diagrams of conventional hydrochemical indexes of different types of groundwater in the study area.

According to the class III water quality criteria in China’s standard for groundwater quality (GB/T 14848-2017) [35], the TDS, total hardness (TH), Na^+^, Cl^−^ and SO_4_^2−^ of the groundwater in the study area exceed the upper threshold by 66.67, 100, 14.81, 48.15 and 51.85%, respectively. The pH varies between 7.0 and 7.7. The pH values of C1, C2, C3 and C4 samples are 7.5 ± 0.2, 7.3 ± 0.1, 7.2 ± 0.1 and 7.1, respectively (Figure 4). The TDS of groundwater in the study area ranges from 548 to 3738 mg/L. The TDS of C1, C2, C3 and C4 samples are 790 ± 207, 1234 ± 186, 1552 ± 240 and 3446 ± 415 m/L, respectively (Figure 4). Similarly, the Ca^2+^, Na^+^, Cl^−^ and SO_4_^2−^ concentrations in the groundwater in the study area range from 130.9 to 541.2 mg/L, 24.04 to 756.2 mg/L, 55.02 to 1986 mg/L and 113.2 to 1178 mg/L, respectively (Figure 4). The pH values of the different types of groundwater sub-groups show inverse relationships with the contents of the main chemical components (Ca^2+^, Na^+^, Cl^−^ and SO_4_^2−^) (Figure 4). As the dissolution processes of evaporites (such as rock salt and gypsum) are independent of the groundwater pH, the Cl^−^ and SO_4_^2−^ in this karst aquifer should not come mainly from the dissolution of evaporites. This suggests that the groundwater in the study area may be polluted by acid wastewater containing hydrochloric acid related to the existence of a large number of oil refining and chemical enterprises in the study area. As the study area mainly contains carbonate aquifer, the chemical acidic wastewater (containing hydrochloric acid and sulfuric acid) sourcing from discharges/spillages from the local chemical industries can be buffered by the dissolution of carbonate rocks, resulting in a nearly neutral pH (pH-buffering effect) and an increase in the Ca^2+^, Mg^2+^, Cl^−^ and SO_4_^2−^ concentrations in karst groundwater.

### 3.3. Hydrogeochemical Analysis of Karst Groundwater Polluted by Acidic Chemical Wastewater

**Figure 5:**
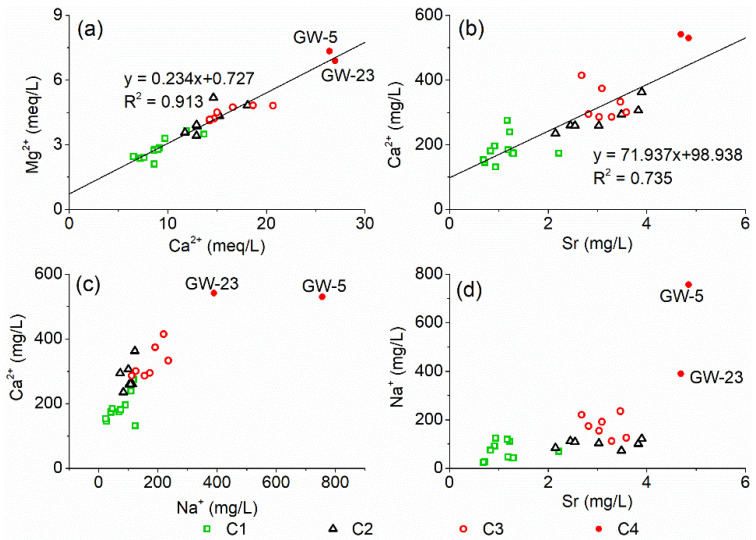
The relationship between the concentrations of Ca^2+^, Mg^2+^, Sr and Na^+^ in groundwater in the study area.

The Ca^2+^ in karst water mainly comes from the dissolution of carbonate (such as calcite and dolomite) or gypsum, while the Mg^2+^ mainly comes from the dissolution of dolomite. There is a significant linear positive correlation between the Mg^2+^ and Ca^2+^ concentrations in the groundwater in the study area (R^2^ = 0.913), and the data points of content are distributed near the straight line with a slope of 0.234 (Figure 5a), which may indicate that the Ca^2+^ and Mg^2+^ mainly come from the dissolution of carbonate in this study.

The significant linear positive correlation between Ca^2+^ and Sr concentrations indicates that Sr^2+^ may come from the dissolution of calcium bearing minerals in karst aquifer (Figure 5b). Furthermore, there is a significant positive correlation between Na^+^ and Ca^2+^, Sr (Figure 5c,d), which shows that the changes of Na^+^ as well as Ca^2+^ and Mg^2+^ concentrations in groundwater are not caused by cation exchange. Therefore, the Ca^2+^ and Mg^2+^ in the karst groundwater of the study area come from the dissolution of calcium and magnesium bearing minerals such as calcite and dolomite, rather than the direct anthropogenic input and cation exchange. The significant positive correlation between Na^+^ and Ca^2+^ indicates that such a high concentration of Na^+^ in the groundwater may mainly come from NaCl in acidic wastewater.

Generally, Mg^2+^ in karst groundwater is derived from the corrosion of dolomite minerals by the carbonic acid (H_2_CO_3_) formed mainly by soil CO_2_ dissolution. The milligram equivalence ratio of Mg^2+^ to HCO_3_^−^ is 1:2.

CO_2_ + H_2_O = H_2_CO_3_ (1)

CaMg(CO_3_)_2_ + 2H_2_CO_3_ = Ca^2+^ + Mg^2+^ + 4HCO_3_^−^ (2)

**Figure 6:**
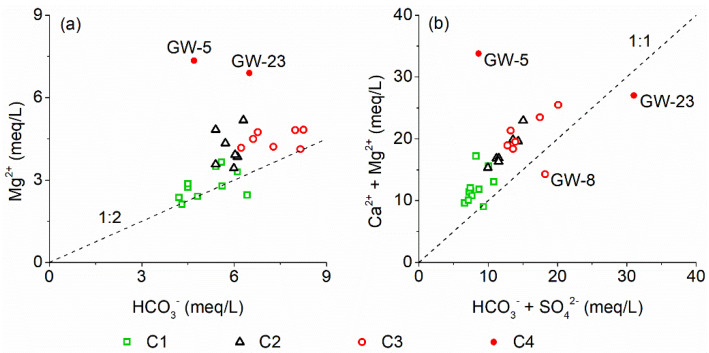
Relationship between Mg^2+^ and HCO_3_^−^, Ca^2+^ + Mg^2+^ and HCO_3_^−^ + SO_4_^2−^ in groundwater in the study area.

However, most groundwater samples in the study area have an Mg^2+^/HCO_3_^−^ milligram equivalence ratio above the equilibrium line that has a slope of 1/2; especially, C4 samples that have high Cl^−^ concentrations (Figure 6a). This indicates that Mg^2+^ in karst groundwater is derived not only from the carbonate weathered by H_2_CO_3_, but also the carbonate corroded by hydrochloric acid.

In addition, almost all the ion data points for groundwater samples in the study area sit above the milligram equivalence line: (Mg^2+^ + Ca^2+^)/(HCO_3_^−^ + SO_4_^2−^) = 1 (Figure 6b). In other words, even if Ca^2+^ is contributed by the dissolution of gypsum, the HCO_3_^−^ from the carbonate weathered by H_2_CO_3_ is still insufficient to balance Mg^2+^ and Ca^2+^. The above analysis has shown that Mg^2+^ and Ca^2+^ are not derived from anthropogenic input and cation exchange; thus, it further indicates the existence of carbonate dissolution by hydrochloric acid wastewater sourcing from discharges/spillages from the local chemical industries.

**Figure 7:**
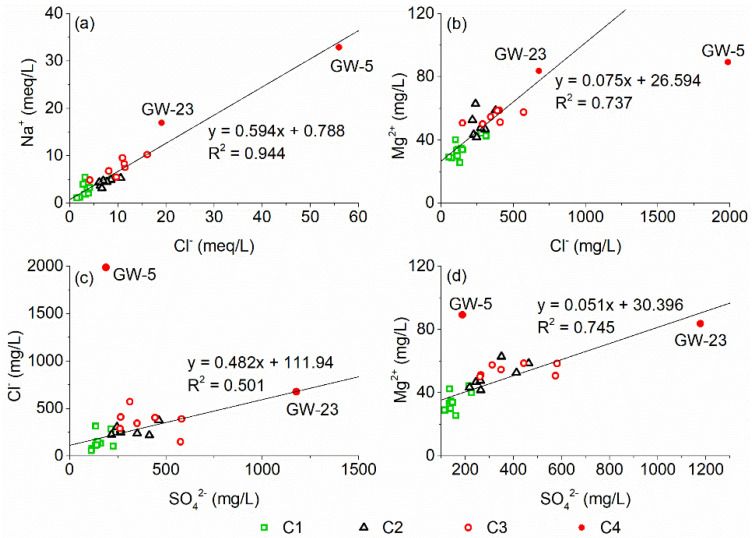
The relationship between the concentrations of Na^+^, Mg^2+^, Cl^−^, SO_4_^2−^ in groundwater in the study area.

The Cl^−^ and Na^+^ in groundwater usually come from halite (NaCl) dissolution, atmospheric precipitation and anthropogenic inputs [36]. Furthermore, the excess of Na^+^ is attributed from silicate (e.g., albite) weathering as well as cation exchange [37]. There is a significant positive correlation between Na^+^ and Cl^−^ in the groundwater in the study area (Figure 7a), indicating that they may have similar sources or chemical behaviors. Since the Cl^−^ concentrations in the groundwater are significantly higher than in the rainwater, atmospheric deposition is not considered to be a major source of Cl^−^ for the karst aquifer in the study area. The Na^+^/Cl^−^ molar ratio of groundwater in the study area is close to 0.594 (Figure 7a), which means higher Cl^−^ concentrations with respect to Na^+^ than expected from the theoretical 1:1 halite dissolution line. This suggests that Cl^−^ can be attributed from anthropogenic inputs rather than merely NaCl dissolution. Meanwhile, there is a strong positive correlation between Cl^−^ and Mg^2+^ (Figure 7b), confirming that the karst groundwater in the study area is polluted by acidic wastewater containing NaCl and hydrochloric acid (HCl), which promotes the weathering and dissolution of carbonate. The reactions are as follows:

CaMg(CO_3_)_2_ (dolomite) + 2HCl = Ca^2+^ + Mg^2+^ + 2Cl^−^ + 2HCO_3_^−^ (3)

CaCO_3_ (calcite) + HCl = Ca^2+^ + Cl^−^ + HCO_3_^−^ (4)

In addition, there is a strong positive correlation between Cl^−^ and SO_4_^2−^ in the groundwater of the study area (Figure 7c), which suggests that the acidic wastewater contains sulfuric acid. At the same time, Mg^2+^ and SO_4_^2−^ also show a strong positive correlation (Figure 7d), suggesting the dissolution of carbonate by sulfuric acid (H_2_SO_4_). The reactions are as follows:

CaMg(CO_3_)_2_ (dolomite) + H_2_SO_4_ = Ca^2+^ + Mg^2+^ + SO_4_^2−^ + 2HCO_3_^−^ (5)

2CaCO_3_ (calcite) + H_2_SO_4_ = 2Ca^2+^ + SO_4_^2−^ + 2HCO_3_^−^ (6)

## 4. Conclusions

The total hardness, TDS, Cl^−^ and SO_4_^2−^ concentrations of karst groundwater in the study area are generally high, and the total hardness of all groundwater and TDS, Cl^−^ and SO_4_^2−^ of nearly half of the groundwater samples exceed the quality indexes of class III water in the Chinese standard for groundwater quality (GB/T 14848-2017) [35]. The hydrochemical types of karst groundwater are mainly transforming from bicarbonates to chlorides and sulfates, and the key reason is the pollution of karst groundwater to different degrees by hydrochloric acid and sulfuric acid wastewater leaked from chemical enterprises in the study area. Thus, chemical acidic wastewater is the primary source of Cl^−^ and SO_4_^2−^ in the karst groundwater. Hydrochloric acid and sulfuric acid in wastewater can promote the dissolution of carbonates, resulting in significant positive correlations between Ca^2+^, Mg^2+^, Sr, Cl^−^ and SO_4_^2−^. Those indicators also show significant negative correlations with pH, which is hydrogeochemical evidence of karst groundwater pollution by acidic wastewater. Due to the dissolution of carbonate rocks, the pH of karst groundwater is buffered and nearly neutral. More in-depth studies (such as environmental isotope tracing technology) are important to verify and quantify the pollution sources. Furthermore, reasonable treatment methods should be developed to reduce the negative impacts of discharges/spillages from the local chemical industries on karst aquifer.
